# Individualized strategies to target specific mechanisms of disease in malignant melanoma patients displaying unique mutational signatures

**DOI:** 10.18632/oncotarget.4545

**Published:** 2015-07-25

**Authors:** Soraya Curiel-Olmo, Almudena García-Castaño, Rebeca Vidal, Helena Pisonero, Ignacio Varela, Alicia León-Castillo, Eugenio Trillo, Carmen González-Vela, Nuria García-Diaz, Carmen Almaraz, Thaidy Moreno, Laura Cereceda, Rebeca Madureira, Nerea Martinez, Pablo Ortiz-Romero, Elsa Valdizán, Miguel Piris, José Vaqué

**Affiliations:** ^1^ Cancer Genomics Group, IDIVAL, Instituto de Investigación Marqués de Valdecilla, Santander, Spain; ^2^ Oncology Service, Hospital Universitario Marqués de Valdecilla, Santander, Spain; ^3^ Department of Pharmacology, University of Cantabria (UC), Santander, Spain, and Centro de Investigación Biomédica en Red de Salud Mental (CIBERSAM), ISCIII, Madrid, Spain; ^4^ Instituto de Biomedicina y Biotecnología de Cantabria (IBBTEC), CSIC, Universidad de Cantabria, Departamento de Biología Molecular, Universidad de Cantabria, Santander, Spain; ^5^ Pathology Service, Hospital Universitario Marqués de Valdecilla, Santander, Spain; ^6^ Plastic Surgery Service Hospital Universitario Marqués de Valdecilla, Santander, Spain; ^7^ Dermatology Service, Instituto I+12, Hospital Universitario 12 de Octubre, Madrid, Spain; ^8^ Department of Pharmacology, Medicine School, Complutense University, Madrid, Spain

**Keywords:** Targeted therapy, melanoma, BRAF, MAPK, somatic mutations

## Abstract

Targeted treatment of advanced melanoma could benefit from the precise molecular characterization of melanoma samples. Using a melanoma-specific selection of 217 genes, we performed targeted deep sequencing of a series of biopsies, from advanced melanoma cases, with a Breslow index of ≥4 mm, and/or with a loco-regional infiltration in lymph nodes or presenting distant metastasis, as well of a collection of human cell lines. This approach detected 3–4 mutations per case, constituting unique mutational signatures associated with specific inhibitor sensitivity. Functionally, case-specific combinations of inhibitors that simultaneously targeted MAPK-dependent and MAPK-independent mechanisms were most effective at inhibiting melanoma growth, against each specific mutational background. These observations were challenged by characterizing a freshly resected biopsy from a metastatic lesion located in the skin and soft tissue and by testing its associated therapy *ex vivo* and *in vivo* using melanocytes and patient-derived xenografted mice, respectively.

The results show that upon mutational characterization of advanced melanoma patients, specific mutational profiles can be used for selecting drugs that simultaneously target several deregulated genes/pathways involved in tumor generation or progression.

## INTRODUCTION

Melanoma is a form of cancer whose incidence is rising each year in the developed world, and is second to leukemia in terms of loss of years of potential life from cancers [1]. Despite recent improvements in mortality rates, current deaths from melanoma are estimated to comprise 85% of all cancers affecting the skin. This is corroborated by the poor survival associated with melanoma when diagnosed at an advanced stage [2]. Therefore, the development of effective therapies is a major challenge in this field.

Molecular diagnostics of cancer have proved that targeted therapies can be effective in many cancer settings, as measured by the recent improvement in cancer survival statistics (World Cancer Statistics 2008; ICD-10 C18–21). The use of EGFR inhibitors in lung cancer [3, 4] and Imatinib in chronic myeloid leukemia (CML) patients [5] are two relevant examples. Targeted therapies in melanoma are mostly directed towards inhibiting MAPK-ERK1/2 signaling (MAPK hereafter), [6]. Mutational analyses have recently enabled the detection of up to 50% of malignant melanomas carrying an activating mutation in *BRAF* [7], and these can now be treated with specific B-RAF inhibitors [8]. In the clinic, this targeted approach, even when used in combination with MEK inhibitors, is of limited benefit to patient survival and, after a period, the cancer reappears aggressively [9–11].

From a molecular perspective, data from Next Generation Sequencing (NGS) show that more mutated genes than initially expected participate in tumorigenesis, including that of melanoma [12–14]. This involves a dynamic process of subclonal competition that eventually dictates multifactorial clinical resistance to B-RAF inhibitors, which is dependent on reactivation of MAPK signaling or other proliferative and/or pro-survival pathways [15–17].

Taking advantage of available melanoma NGS data, we characterized biopsies from advanced melanoma patients and cell lines by studying the presence of somatic mutations in a selected group of genes. We thereby detected unique signatures of mutated genes that are potentially associated with specific inhibitors, and explored the effects of case-specific combinations of the latter *ex vivo* and *in vivo*. Guided by individual mutational profiles, tailored combinations of inhibitors simultaneously targeting MAPK-dependent and MAPK-independent signaling were very efficient at inhibiting aberrant melanoma growth assessed in multiple cell lines, and xenografted tumors and biopsies grown in mice. Thus, specific mutational signatures could guide the design of personalized therapies based on the use of specific combinations of drugs that target case-specific pathogenic signaling mechanisms.

## RESULTS

### A targeted approach to characterizing the mutational status of lesions of advanced melanoma patients

**Figure 1:**
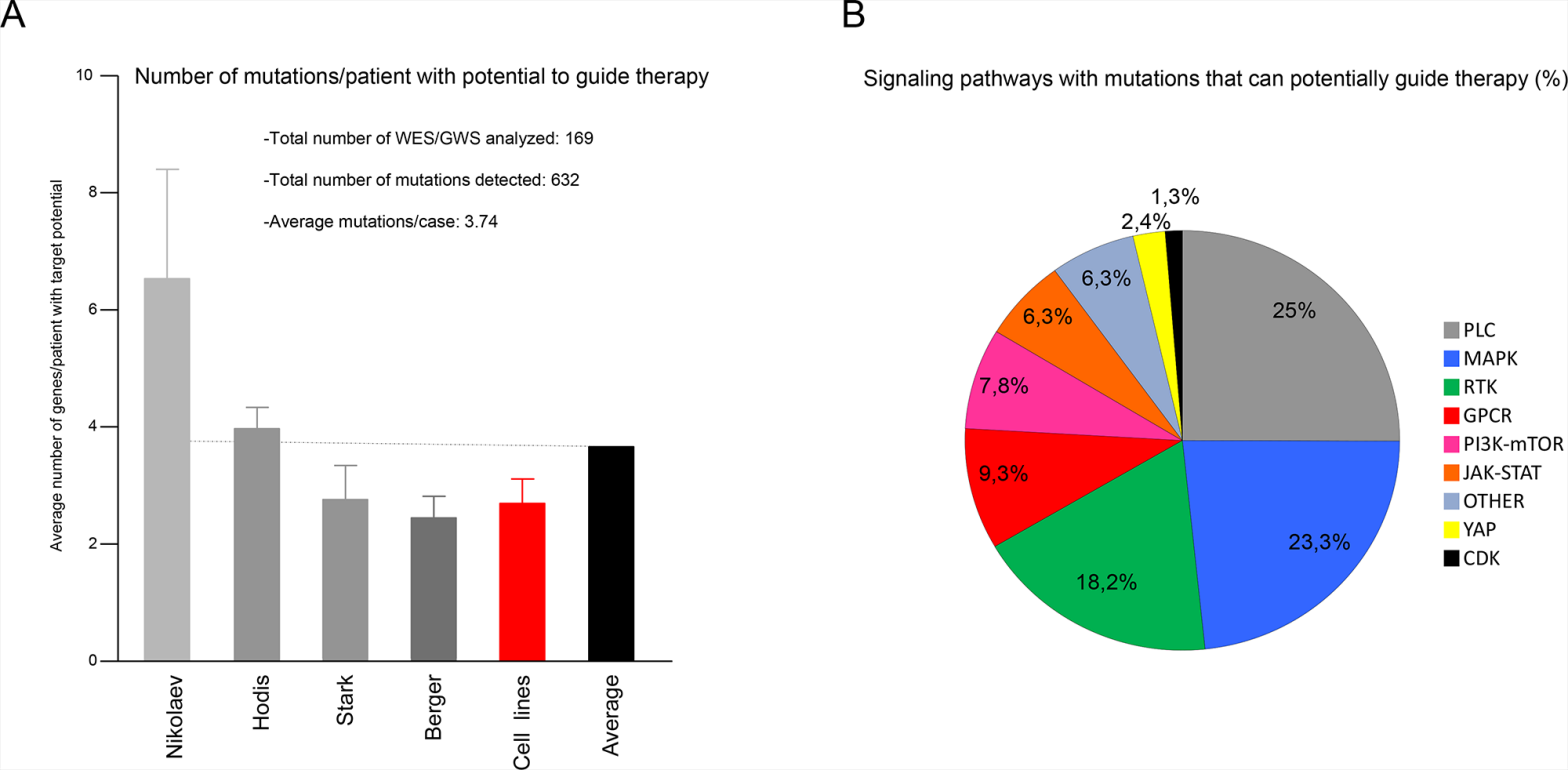
*In silico*-targeted mutational profiling of advanced melanoma patients **A.** Meta-analysis showing the average number of mutated genes per case with the potential to guide targeted therapy. Original mutational data from cell lines (red bar) were obtained from the Cancer Cell Line Encyclopedia website (see Material and Methods); mutational data from patients (grey bars) were obtained from Nilolaev [19], Hodis [18], Stark [14] and Berger [13]; Black bar, shows the average frequency of mutations amongst all data sets. **B.** Percentage of hits in A) involved in the indicated signaling pathway.

To better understand the molecular character of specific melanoma lesions, we set up a targeted mutational study followed by functional analyses (described in Supplementary Figure 1). The genomic design of this study focused on the coding regions of a specific group of 217 genes that had previously been shown to be mutated in melanoma and selected mainly on the basis of their relevance in melanoma and their association with inhibitors of potential clinical use (see Materials and Methods for further explanation). To test this approach our selection of genes was compared *in silico* with the whole genome/exome sequencing (WGS/WES respectively) data already available for 11 advanced melanoma cell lines and 158 human melanomas (see Materials and Methods, [13, 14, 18, 19]). This comparison revealed an average of 3.74 mutated genes that can participate in multiple targetable signaling pathways, including PLC, MAPK, RTKs (receptors with tyrosine kinase activity), PI3K-mTOR and JAK-STAT (Figure 1 and Supplementary Table I). These results prompted us to study advanced melanoma cases (Breslow index ≥4 mm or metastasis) in 18 clinically characterized patients (clinical characteristics summarized in Supplementary Table II) using a targeted primary ultrasequencing approach, followed by secondary validation analysis (see Materials and Methods for further details). By these methods, an average of 3.4 mutated genes were identified in 11 of the 18 patients, enabled the detection of lesion-specific genes such as *BRAF*, *RAC1*, *KRAS*, *HGF* and *MAPK7*, amongst others. Interestingly, there was a wide range of mutation frequencies and combinations, which perhaps reflects the rich and heterogeneous microclonal composition expected in melanoma tumors (400X average depth/mutation; Table I) [20]. Furthermore, actionable mutations such as *BRAF^V600E^* that can guide targeted therapy (using B-RAF inhibitors) were detected in the same melanoma alongside other mutated genes that may also guide therapy (Table I). It is significant that mutations in four patients could not be validated due to limitations of the tissue sample (see Materials and Methods), and that no mutations were identified in three other patients. Thus, this targeted approach could be adopted to identify genomic alterations affecting one or several genes. These may be explored as potential targets for therapy in specific cases of melanoma.

**Table 1:** Validated mutations found in advanced melanoma patients

Patient	Chrom.	Position	Ref_base	Mut_base	Total_Cov	Observed_Freq	Gene_ID	p.Annot
2	Chr4	126370467	G	A	59	0.58	**FAT4**	p.E2766K
2	Chr2	141986902	C	T	83	0.18	**LRP1B**	p.E234K
4	Chr5	167881030	GGA	-	464	0, 53	**WWC1**	p.V861 VE > V
4	Chr5	150923714	T	C	724	0.39	**FAT2**	p.N2325S
4	Chr17	7578490	A	C	884	0.08	**TP53**	p.V147G
5	Chr12	25380269	C	G	177	0, 16	**KRAS**	p.E63D
5	Chr1	9781272	G	C	424	0, 07	**PIK3CD**	p.G593R
6	Chr7	140453136	A	T	39	0, 69	**BRAF**	p.V600E
6	Chr3	3134041	T	A	100	0, 3	**IL5RA**	p.E287D
6	Chr19	15290897	C	T	1200	0, 27	**NOTCH3**	p.G1105S
8	Chr7	6426892	C	T	233	0, 67	**RAC1**	p.P29S
8	Chr7	81346551	G	A	243	0, 61	**HGF**	p.R468C
8	Chr7	140453136	AC	CT	61	0, 54	**BRAF**	p.V600R
8	Chr3	155311800	C	T	145	0, 49	**PLCH1**	p.G104S
8	Chr18	51053024	CC	TT	119	0, 48	**DCC**	p.S1383F
8	Chr5	89910783	C	T	60	0, 4	**GPR98**	p.R52C
8	Chr1	23233289	T	G	76	0, 39	**EPHB2**	p.Y659D
8	Chr13	28886195	C	T	190	0, 32	**FLT1**	p.E1143K
8	Chr18	50432552	C	T	163	0, 37	**DCC**	p.P184L
8	Chr2	170136083	C	T	38	0, 29	**LRP2**	p.G455D
8	Chr11	46406865	G	A	1048	0, 27	**CHRM4**	p.P415S
9	Chr7	31855742	C	T	66	0, 24	**PDE1C**	p.E597K
9	Chr7	140453135	CA	TT	55	0, 11	**BRAF**	p.V600E
12	Chr7	140453136	A	T	142	0, 1	**BRAF**	p.V600E
12	Chr6	32170007	C	T	302	0, 06	**NOTCH4**	p.G1201R
13	Chr2	166905414	C	T	321	0, 15	**SCN1A**	p.G337E
13	Chr5	55247832	G	A	362	0, 12	**IL6ST**	p.L542F
16	Chr16	9858517	C	T	450	0, 06	**GRIN2A**	p.E962K
17	Chr7	140453136	A	T	326	0, 44	**BRAF**	p.V600E
17	Chr4	126239082	C	T	1417	0, 27	**FAT4**	p.L506F
17	Chr17	19285669	C	T	1646	0, 23	**MAPK7**	p.P546S
17	Chr18	50450115	C	T	371	0, 2	**DCC**	p.P246S
18	Chr10	96014751	C	T	651	0, 13	**PLCE1**	p.P1167S
18	Chr2	21233091	G	A	167	0, 08	**APOB**	p.H2217Y
18	Chr13	29001438	C	T	824	0, 08	**FLT1**	p.E432K
18	Chr7	151851165	CC	TT	876	0, 08	**KMT2C**	p.G4069N
18	ChrX	112035176	C	T	420	0, 07	**AMOT**	p.E195K

Patient: Patient number; Chrom: Chromosome number; Position: Genomic location of the mutation in the chromosome; Ref_base: normal nucleotide; Mut_base: mutated nucleotide; Total Cov: Number of reads analyzed at each position; Observed_Freq: Frequency of mutation; Gene_ID: Gene name; pAnnot: Aminoacid change.

### Effects of specific targeted therapy guided by mutational signature

**Figure 2:**
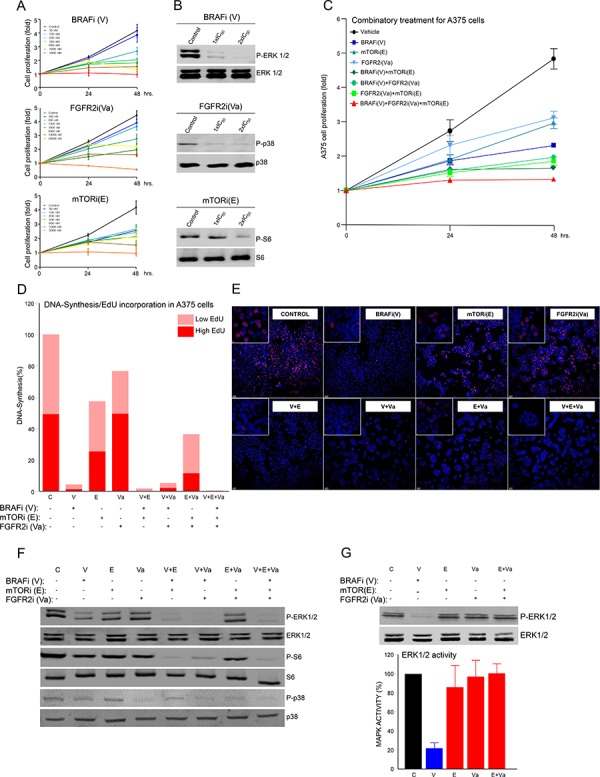
Effects of specific targeted therapy guided by mutational signature **A.** Proliferation analysis of A375 cells at 0, 24 and 48 h. Cells were seeded in 96-well plates and treated with the indicated concentrations of each inhibitor: B-RAFi (V: Vemurafenib), FGFR2i (Va: Vargatef), and mTORi (E: Everolimus). **B.** Western blots using whole cell lysates from starved A375 cells incubated for 1 h with control vehicle (DMSO) or the indicated concentration of each inhibitor. The figure shows a representative experiment using P-ERK1/2, ERK1/2, P-p38, p38, P-S6 and S6 antibodies, as indicated. **C.** Proliferation analysis of A375 cells in the same conditions as in A), but incubated with control vehicle (DMSO) or the IC_50_ concentration of the indicated inhibitor alone (blue lines), or in a double (green lines) or triple combination (red line). *N* = 6. Error bars show the SEM. **D.** DNA synthesis using Click-iT^®^ EdU in exponentially growing A375 cells seeded in an 8-well glass and incubated for 48 h with control vehicle (DMSO) or the indicated inhibitor or combination of inhibitors, as in C). Graph bars show percentage of low (clear red) or high (intense red) EdU-stained cells in three photographic fields from a representative experiment. **E.** Representative pictures of each treatment condition showing the nucleus of the total number of cells (blue dots) and EdU-positive cells (red dots). **F** and **G.** Western blots of whole cell lysates of the indicated cells. Cells were starved overnight and incubated for 1 h with control vehicle (DMSO), or the indicated inhibitor, or a combination of inhibitors under the same conditions as in C). Figures show representative experiments using P-ERK1/2, ERK1/2, P-p38, p38, P-S6 and S6 antibodies, as indicated. Bar graphs show the values of three independent experiments in G). Error bars indicate the SEM.

To explore how to use mutational data to design targeted therapies based on specific mutational characterizations, the functional effects of specific combination therapies were studied in advanced melanoma cell lines with known mutational profiles (Supplementary Table III). Taking A375 advanced melanoma cells as an example, we detected and validated mutations in *BRAF*, *FGFR2* and *mTOR* that could reasonably be expected to associate with Vemurafenib (BRAFi (V), hereafter), Vargatef (FGFR2i (Va)) and Everolimus (mTORi (E)). Exponentially growing A375 cells were incubated with increasing concentrations of each inhibitor. This caused a concentration-dependent reduction in cell proliferation from which the IC_50_ of each inhibitor was calculated (Figure 2A and Supplementary Table III). These concentrations were used for subsequent experiments. Next, the mechanistic effects of treatment with each inhibitor (using IC_50_ values in each case) were analyzed in A375 cells that had been serum-starved to provoke the inhibition of the intended mutation-associated downstream signaling. These were assessed by western blot using P-ERK1/2, P-p38 and P-S6 antibodies (Figure 2B).

To discover more about the biological effects of multiple combinations of these inhibitors on proliferation, A375 cells were incubated with IC_50_ concentrations of BRAFi, FGFR2i and mTORi in single, double or triple combinations (blue, green and red lines, respectively, in Figure 2C). The combinatorial treatments were more effective at reducing melanoma cell growth than the monotherapies. The triple combination was the most efficient, and had no non-specific cytotoxic effects (Figure 2C and 2E). These results were confirmed using DNA synthesis as an alternative read-out (Figure 2D and 2E). Thus, under these conditions, a combination of inhibitors guided by a specific mutational signature, simultaneously targeted multiple signaling mechanisms controlling the growth of A375 cells. Analyzing the mechanistic effects of these drug combinations on their associated signaling pathways in this system, showed that treatment with BRAFi inhibited MAPK signaling. However, treatment of A375 cells with the inhibitors mTORi and FGFR2i, alone or in combination (E+Va), had no such effect (Figure 2F and 2G), despite being very effective at inhibiting cell proliferation and DNA synthesis (Figures 2C, and 2E). Thus, using genetically defined inhibitors in this system we can specifically target a combination of MAPK-dependent (V) and MAPK-independent (E+Va) signaling mechanisms that control the malignant growth of A375 melanoma cells. This observation was not confined to these cells and more examples of specific mutational signatures guiding effective combinatorial therapies comprising MAPK-dependent and MAPK-independent mechanisms in other human advanced melanoma cell models are shown in Supplementary Figures 2, 3 and 5.

### Increased effects of targeted therapy against an appropriate mutational background

**Figure 3:**
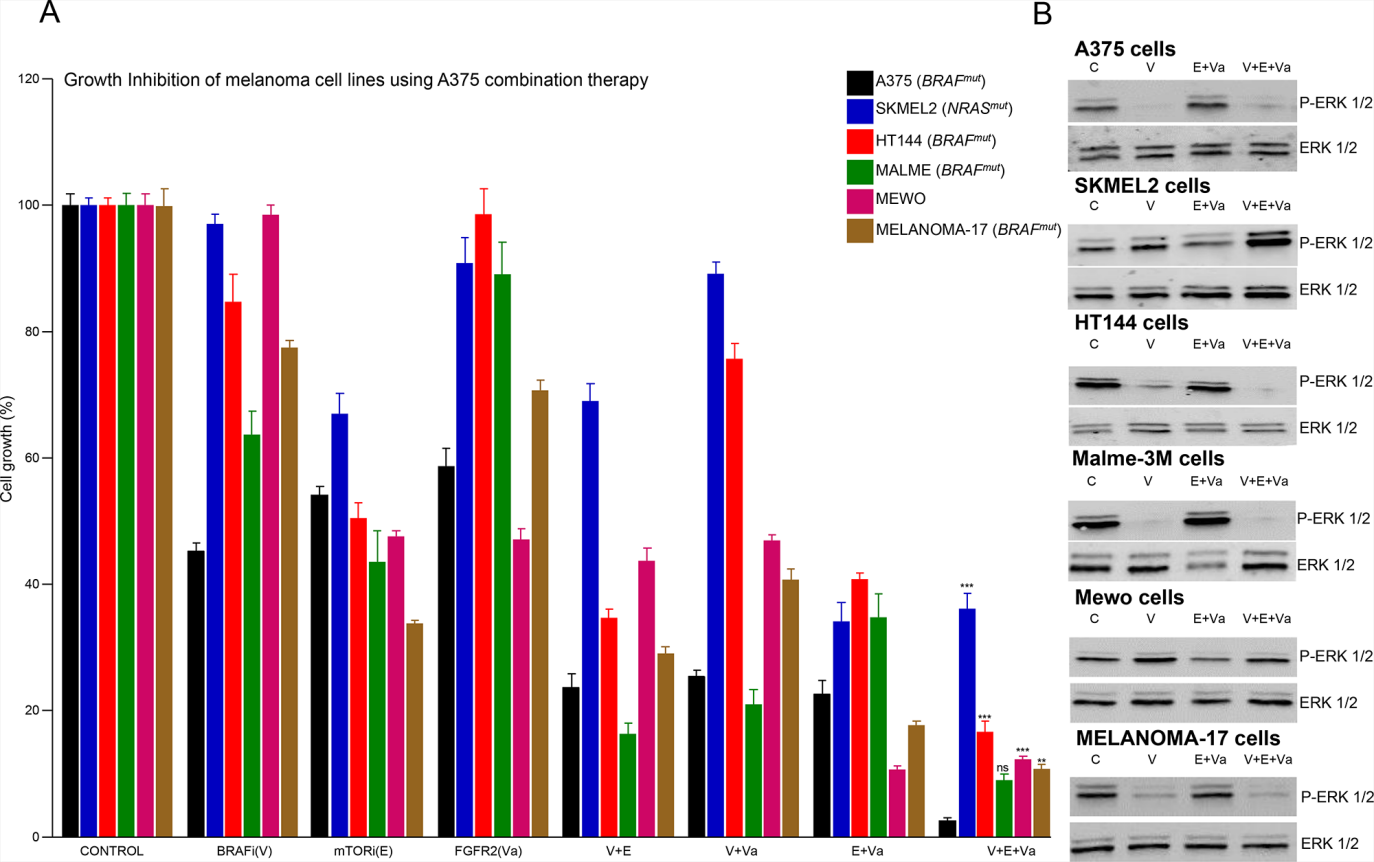
Increased effects of targeted therapy against an appropriate mutational background **A.** Proliferation analysis of exponentially growing A375 (BRAF+), SKMEL2 (NRAS+), HT144 (BRAF+), MALME (BRAF+), MEWO and MELANOMA17 (BRAF+) cells treated with vehicle (DMSO) or the IC_50_ concentration (calculated for A375 cells) of the indicated inhibitor alone, in a double or triple combination for 48 h. *N* = 6. Error bars show the SEM. **B.** Western blots of whole cell lysates of the indicated cells. Cells were starved overnight and incubated for 1 h with control vehicle (DMSO), or the indicated inhibitor, or a combination of inhibitors under the same conditions as in A). Figure shows a representative experiment.

As part of a heterogeneous network of aberrant intracellular signaling, multiple deregulated pathways can participate in the mechanistic control of melanoma growth (Figure 1). We examined whether a combination therapy designed for a specific mutational signature could be more effective when used against a genetically appropriate background. A group of melanoma cell lines harboring unique mutational signatures (Supplementary Table III) was treated in parallel with the genetically defined inhibitors for A375 cells, BRAFi, FGFR2i and mTORi, alone or in combination. In general, each treatment was more effective in A375 cells than in the other melanoma cell lines, with the possible exception of mTORi used alone (Figure 3A). The triple drug combination treatment (V+E+Va) simultaneously targeting MAPK-dependent and MAPK-independent proliferation mechanisms produced greater inhibition of A375 cell growth than the others (Figure 3A and 3B). Likewise, other specific treatments based on the combination of genetically defined inhibitors in other melanoma cell lines showed a stronger effect than that of A375 cells (Supplementary Figure 4A and 4B). More examples of specific mutational signatures guiding more effective combination therapies when used against an appropriate mutational background are shown in Figure 5E and Supplementary Figure 5C. In summary, based on specific mutational signatures, a specific treatment consisting of a combination of genetically defined inhibitors may have stronger anti-melanoma activity when used against an appropriate genomic background.

### *In vivo* effects of a targeted therapy that combines MAPK-ERK-dependent and MAPK-ERK-independent mechanisms of inhibition

**Figure 4:**
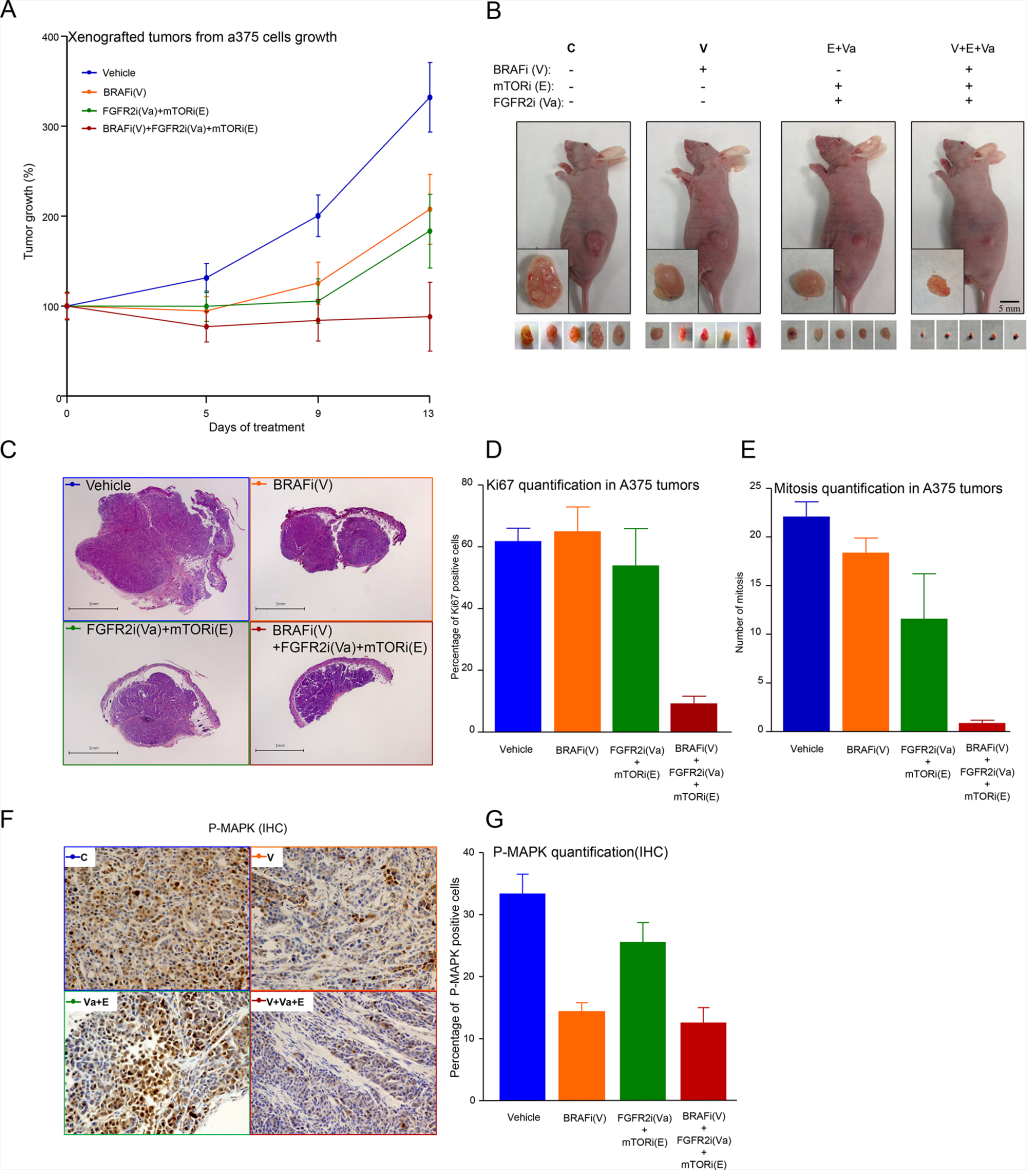
*In vivo* effects of a targeted therapy combining MAPK-ERK-dependent and MAPK-ERK-independent mechanisms of inhibition **A.** Xenografted tumor growth-derived A375 cells injected subcutaneously in 48 BALB/C nude mice. Tumor size was monitored until a volume of 100 mm^3^ was obtained, whereupon mice were assigned to four treatment groups: 1) Control (DMSO, blue line); 2) BRAFi (V) (orange line); 3) FGFR2i (Va) + mTORi (E) (green line); and 4) BRAFi (V) + FGFRi (Va) + mTORi (E) (red line). Mice were treated daily as indicated (see Materials and Methods for further details) and tumor volumes were measured until day 13, at which point the experiment was ended. Data were obtained from the 12 control, 11 (V), 7 (Va+E) and 10 (V+Va+E) mice that survived the entire process. Error bars indicate the SEM. **B.** Representative pictures illustrating the effects of the indicated treatment on the xenografted tumors that had been resected or were still in the mice. **C.** H&E staining of representative tumor sections from five representative mice for each treatment condition. Tumor sections were analyzed for Ki67-positive staining **D.** or by the number of mitoses **E.** Data are averages of five section cuts in each mouse. Error bars indicate the SEM. **F.** Immunohistochemical (IHC) analysis in tumors corresponding to the indicated treatment, as in C), using an anti-phospho ERK antibody stain. **G.** Tumor sections were analyzed for phosphor-ERK-positive staining. Results are the averages of five section cuts per mouse. Error bars indicate the SEM.

**Figure 5:**
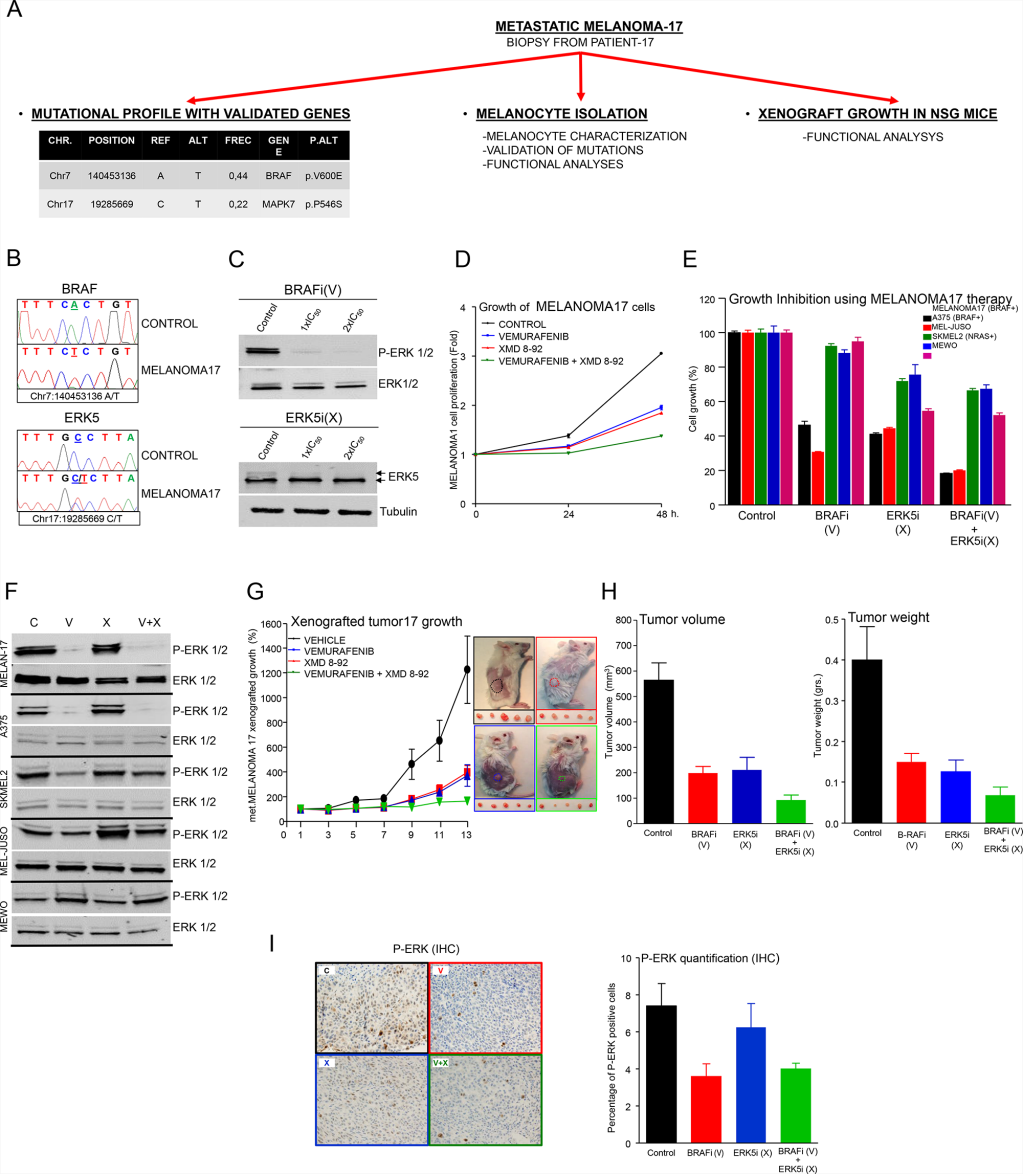
A pre-clinical example of targeted therapy guided by a specific mutational signature in melanoma patient 17 **A.** Schematic representation of the work performed with a freshly resected biopsy from patient 17. **B.** Sanger sequencing of *BRAF* (above) and *MAPK7* (*ERK5*; below) oncogenes in genomic DNA from control cells or isolated melanocytes from patient 17 (MELANOMA17 cells). **C.** Western blots of whole-cell lysates from starved MELANOMA17 cells incubated for 1 h with control vehicle (DMSO) or the indicated concentration of each inhibitor BRAFi (V: Vemurafenib) or ERK5i (X: XMD-8–92). The figure shows a representative experiment using P-ERK1/2, ERK1/2, ERK5 and tubulin antibodies, as indicated. **D.** Proliferation analysis of MELANOMA17 cells at 0, 24 and 48 h. 3 × 10^3^ cells/well were seeded in 96-well plates and treated with control (DMSO) (black line), or an IC_50_ concentration of B-RAFi (V) (blue line) or ERK5i (X) (red line), alone or in combination (green line). *N* = 6. Error bars show SEM. **E.** Proliferation analysis of MELANOMA17, A375, MEL JUSO, SKMEL2, and MEWO cells at 0, 24 and 48 h, under the same conditions as in D). *N* = 6. Error bars show the SEM. **F.** Western blots using whole cell lysates of the indicated cells. Cells were starved overnight and incubated for 1 h with control vehicle (DMSO), or the indicated inhibitor, or a combination of inhibitors using the same concentrations as in E). Representative experiment using anti-P-ERK1/2 and anti-ERK1/2 antibodies. **G.** Tumor growth derived from 2-mm^3^ MELANOMA17-derived tumor fragments implanted subcutaneously in 30 NSG mice (Jackson Laboratories). Tumors were monitored until they attained a volume of 100 mm^3^, whereupon mice were assigned to four comparable treatment groups: 1: Control (DMSO, black line), 2: BRAFi (V) (blue line), 3: ERK5i (X) (red line) and 4: BRAFi (V) + ERK5i (X) (green line). Mice were treated daily as indicated (see Materials and Methods for further details) and tumor volumes were monitored until day 13, at which point the experiment was ended. Data were obtained from five survivor mice from each treatment group. Error bars indicate the SEM. The figure shows a representative image from treated tumors that were still in the mice (above) or had been freshly resected (below). **H.** Bar graph of average changes in tumor volume (left) and mass (right) for each treatment condition. *N* = 5. Error bars indicate the SEM. **I.** Examples of IHC analysis of tumors corresponding to the treatment indicated in D) using anti-phospho ERK antibody staining. Bar graphs show results for tumor sections analyzed for phosphor-ERK-positive staining. Data are the averages of five section cuts per mouse. Error bars indicate the SEM.

To study the *in vivo* effects of targeted therapy oriented by a specific mutational profile, xenografted tumors from A375 melanoma cells were generated in BALB/C mice (nu^−/−^/nu^−/−^). Once grown to a volume of approximately 100 mm^3^, tumors were assigned to four comparable groups and treated daily with vehicle, a MAPK-dependent inhibitor (BRAFi), a MAPK-independent combination of inhibitors (FGFR2i+ mTORi), or a triple combination of the latter (BRAFi+FGFR2i+mTORi). As shown in Figure 4A and 4B, both treatments used independently reduced tumor growth to a similar extent. However, the triple combination (V+Va+E) proved most effective at reducing melanoma growth in this system. Once the experiment was finished, the remaining growth potential of these tumors was characterized by studying Ki67 and the mitotic index in tumor sections. As might be expected, a marked decrease in both proliferation markers in those tumors treated with the combination of MAPK-dependent and MAPK-independent inhibitors (Figure 4C-4G) was observed, thereby confirming *in vivo* our previous findings in cultured cells (Figures 2D, 2E, and 5).

### A pre-clinical example of targeted therapy guided by a specific mutational signature in patient 17

These findings were challenged by integrating the study of a freshly resected biopsy from patient 17 (Table I and Supplementary Table II) in the working pipeline (illustrated in Figure 5A and Supplementary Figure 1). First, a fragment of the biopsy was characterized which enabled the detection of somatic mutations in the *BRAF* and *MAPK7* (*ERK5* hereafter) genes (Table I and Figure 5A), which were associated with specific inhibitors like Vemurafenib (BRAFi (V)) and XMD-8–92 (ERK5i (X)).

Second, freshly isolated melanocytes (MELANOMA17 cells hereafter) were also inspected for the presence of mutations of *BRAF* and *ERK5* (Figure 5B) and for the expression of well-known melanoma markers such as S100A and MCSP (Supplementary Figure 6A and 6B). Once characterized, MELANOMA17 cells were incubated with increasing concentrations of BRAFi and ERK5i, and the IC_50_ of each was calculated (Supplementary Table III). Treatment with BRAFi and ERK5i inhibited B-RAF and ERK5-dependent signaling, assessed by western blot, in starved MELANOMA17 cells (Figure 5C). Simultaneous treatment with both inhibitors was more effective at reducing MELANOMA17 cell proliferation than either inhibitor alone (Figure 5D). Furthermore, and consistent with our previous observations in multiple cell lines, the combination treatment (BRAFi+ERK5i) was also more effective in cells with an appropriate mutational signature (MELANOMA17 cells) when compared with a panel of other melanoma cell lines (Figure 5E), with the possible exception of A375 cells, which were highly sensitive to treatment with the BRAFi dose used (compare the IC_50_ values for MELANOMA17 with those of A375 cells in Supplementary Table III). This combination therapy also consisted of MAPK-dependent (BRAFi) and MAPK-independent (ERK5i) mechanisms, which successfully suppressed the aberrant growth of MELANOMA17 cells (Figure 5F).

Third, another fragment of the biopsy was implanted in NSG mice and allowed to grow until four tumor-comparable groups of mice could be established. The groups of mice were then treated with vehicle, BRAFi, ERK5i, or a combination of the two (V+X). When used separately, the two inhibitors were able to suppress xenografted MELANOMA17 tumor growth to a similar extent, but growth inhibition was more effective when used in combination (Figure 5G-5I).

Thus, it is possible to study the effects of personalized therapies, guided by targeted mutational profiling of advanced melanoma patients, in pre-clinical *ex vivo* and *in vivo* models using freshly resected material from each lesion.

## DISCUSSION

Metastatic melanoma provides an instructive example of the development of rationalized therapies guided by molecular diagnostics. Mechanistically, targeted therapy mainly involves the inhibition of the MAPK signaling pathway by using BRAF or MEK inhibitors, alone or in combination [21], as suggested by: A) activating mutations in BRAF and NRAS oncogenes in 48% and 15% of all diagnosed melanomas, respectively [22]; and B) multiple MAPK reactivation mechanisms that confer resistance to BRAF inhibitors [23–25]. This has improved the clinical management of those patients with mutated *BRAF*, whereby targeted inhibition of aberrant MAPK signaling can increase their OS by up to 11.4 months, although, from a different perspective, it still offers a limited benefit to these patients [10, 26, 27]. To explain this, we can hypothesize that, as part of an intricate network of transforming mechanisms in melanoma, this disease simultaneously uses multiple oncogenic mechanisms, such as, for example, PI3K, MET and GNAQ [28, 29], that, along with MAPK signaling, act as mechanistic drivers of the disease and promote progression or resistance to therapy. In this regard, data from genetically defined melanoma models now show that a rational combination of MAPK and PI3K inhibitors can improve the effects of therapy when used against specific genomic backgrounds [30]. Moreover, patients with specific mutations gained greater benefits when treated with immunotherapy [31, 32]. Thus, better characterization of advanced melanoma lesions could improve our ability to treat this disease through the use of specific therapies that simultaneously target multiple signaling mechanisms.

From a molecular perspective, melanoma is a very heterogeneous disease in which up to 1, 500 somatic mutations may be harbored in the coding exons of a single lesion [13]. This work studies mutations in 217 genes previously shown to be mutated in melanoma [13, 14, 18, 19, 33, 34] *in silico* by comparing them with mutations in 11 cell lines and 158 human melanomas, and *ex vivo* by characterizing 18 lesions from advanced melanoma patients. Under all conditions, genes like *BRAF*, *RAC1*, *FGFR2* and *IL6R* were mutated at varying frequencies, occurring as part of unique mutational signatures comprising specific combinations of mutated genes that have the potential to participate in multiple signaling pathways and to be associated with specific inhibitors. Functionally, the effects of combination therapies guided by specific mutational signatures were analyzed in multiple melanoma cells harboring unique mutational signatures. Those treatments that simultaneously targeted MAPK-dependent and MAPK-independent signaling were most effective at reducing melanoma growth both *ex vivo* and *in vivo*. These observations can be aligned with work from other laboratories, showing that to promote transformation in melanocytes, aberrant MAPK-signaling elicited by BRAF or NRAS oncoproteins requires the active collaboration of other oncogenes, such as *PI3K*, *RAL-GDS*, *GNAS* or *C-MYC*, that can participate in alternative signaling pathways [35–38]. Thus, a combination of genetically defined inhibitors targeting multiple signaling pathways could be more effective against specific cases of malignant melanoma. We might expect targeting well-known melanoma mechanisms to affect the growth of melanoma cells in general, and this can indeed be observed in our data. Nevertheless, combination therapies guided by specific mutational signatures were most effective when used against an appropriate mutational background. Furthermore, different *BRAF*-mutated cell lines, each with an individual mutational signature, had different sensitivities to BRAF inhibition (Supplementary Table III). Finally, our data strongly suggest that combining several mutationally selected inhibitors can specifically block important mechanisms that participate in the control of aberrant melanoma cell growth and in the finely tuned cellular decision to activate DNA synthesis (Figures 2B, 2C, 2E, 4A, 4D and 4E). This rules out the possibility that the results were a consequence of nonspecific cytotoxic activities.

Starting with freshly resected material from a metastatic lesion (patient 17) and trying to match the timing with the clinic, a validated mutational profile was obtained within two weeks of resection. This data enabled the study of a combination therapy based on inhibitors with MAPK-dependent (BRAFi) and MAPK-independent (ERK5i) mechanisms of action in isolated MELANOMA17 cells and in xenografted tumors grown in mice. These gave the best results when combinatorial approaches were used. Of course, this study provides just one example of what targeted characterization of specific lesions might offer by way of diagnostic possibilities for human melanoma in the near future. Considering its potential applicability in routine clinical practice, this approach would require several limitations to be overcome. This would entail: 1) establishing efficient protocols to collect, manipulate and characterize specific lesions that are representative of the various steps of the disease; 2) managing the toxicity due to drug combinations; and 3) dealing with tumor heterogeneity and interactions with the immune system that may be responsible for the eventual resistance acquired after combination treatments. However, there is much scope for studying novel strategies for targeted therapy following a molecular rationale, particularly in a disease like advanced melanoma that offers a limited prospect of survival to patients who are suffering from it (Supplementary Figure 7).

In summary, by adopting targeted approaches we can envisage working with specific signatures of mutated genes that can: 1) help characterize individual lesions in advanced melanoma patients; 2) guide the use of specific inhibitors rationally combined in individualized therapies to target case-specific mechanisms of melanocytic transformation. In this work, a rational combination of genetically defined inhibitors simultaneously targeting MAPK-dependent and MAPK-independent signaling mechanisms showed improved biological outcomes with respect to the malignant growth of specific advanced melanomas.

## MATERIALS AND METHODS

### Cells and reagents for tissue culture

Eight human advanced melanoma cell lines were used. A375 (CRL-1619™ ), SK-MEL-28 (HTB-72™), SK-MEL-2 (HTB-68™), MALME-3M (HTB-64™), MEWO (HTB-65™) and HT-144 (HTB-63™) cells were obtained from the American Type Cell Collection (ATCC, Rockville, MD). MEL-JUSO (ACC 74) was obtained from the German Collection of Microorganisms and Cell Cultures (DSMZ, Braunschweig, Germany). Genomic data from these cells, including those of the somatic mutations detected in this study, are publicly available at the Broad-Novartis Cancer Cell Line Encyclopedia website (CCLE:http://www.broadinstitute.org). MELANOMA17 cells were established from a primary biopsy sample, as explained in the Supplementary Methods. Commercial cell lines were cultured as recommended by ATCC or DSMZ and incubated with inhibitors, as described in the Supplementary Methods.

### Cell proliferation and DNA synthesis assays

Cells growing exponentially to approximately 50% confluence in T96 well plates were incubated with the specific inhibitors while keeping the total amount of DMSO (0.5%) constant. Cellular proliferation was evaluated using AlamarBlue reagent (Life Technologies) and colorimetric changes were quantified using the Synergy™ HTX Multi-Mode Microplate Reader (Biotek). To assess the effects on DNA synthesis, cells were grown in a Millicell EZ SLIDE 8-well glass (Merck Millipore, PEZGS0816) and after treatment with specific inhibitors were incubated for a further 2 h with Click-iT^®^ EdU (Alexa Fluor^®^ 594 Imaging Kit; Life Technologies, C10339). Immediately afterwards, cells were prepared for microscopy following the manufacturer's specifications (see Supplementary Methods for further explanation). Cell images were captured with a Nikon A1R confocal microscope with Plan Apo 10x/0.45NA and Plan ApoVC 60x/1.40NA objectives.

### Genomic DNA samples

Matched tumoral and non-tumoral material was obtained from 18 patients diagnosed with advanced melanoma and who were being monitored by the Oncology Department of the Hospital Universitario Marqués de Valdecilla (HUMV; see clinical characteristics in Table I). Tumoral DNA samples were obtained from freshly frozen tissue samples taken at the time of diagnosis, and matched non-tumoral DNA was extracted from saliva or peripheral blood neutrophils. We designed an intra-subject observational study of patients diagnosed with advanced melanoma and with a Breslow index of ≥4 mm, and with a loco-regional infiltration in lymph nodes or presenting distant metastasis. The study, the patient information sheet, and the informed consent form were approved by the Ethics Committee of the HUMV.

### Enrichment library design, preparation, sequencing and variant calling

Genomic DNA samples were processed using the Qubit^®^ dsDNA BR Assay Kit (Life Technologies) and quantified using Qubit 2.0 apparatus (Life Technologies). The DNA enrichment library was prepared using a specifically designed HaloPlex Target Enrichment System Kit for this melanoma study (Design ID: 00912-1339502780, Agilent Technologies) following the manufacturer's instructions. The design focused on the coding regions of a group 217 genes known to be mutated in melanoma, and which were selected because they were: A) genes of known relevance in melanoma, including BRAF, NRAS [7], and EGFR [18, 19]; B) genes that may be associated with pharmacological inhibitors of potential clinical use, such as FGFR2, KIT and ERBB4 [18, 20, 21]; and C) genes that may be involved in chromatin architecture (ARID1A and DNMT3A [14]), intracellular signaling (MEK1 [22]), or transcription (NFATC2 [22]). Briefly, 400 ng of genomic DNA was digested with the specific cocktail of restriction enzymes provided in the kit. Digested DNA was then hybridized to a probe for target enrichment, indexed and captured. Each DNA was then amplified by PCR at Tm = 60°C, for 18 cycles, using a Herculase II Fusion Enzyme kit (Agilent Technologies). Next, amplified target libraries were purified using an Agencourt AMPure XP Kit (Beckman Coulter Genomics), following the manufacturer's guidelines, and quantified with Qubit 2.0 apparatus (Life Technologies), using the Qubit^®^ dsDNA HS Assay Kit (Life Technologies). They were also analyzed in parallel by capillary electrophoresis in a 2100 Bioanalyzer (Agilent Technologies), using High Sensitivity DNA reagents and chip Kits (Agilent Technologies). Libraries were sequenced at the Instituto de Medicina Genómica (IMEGEN, Valencia University, Spain) with a MiSeq Personal Sequencer (Illumina). The process of somatic mutation identification described in the Supplementary Methods.

### Somatic mutation identification

Sequencing data were aligned against the human reference genome (hg19) using the BWA aligner [39]. The alignment was refined using SAMTOOLS fixmate (PMID: 19505943) and PICARD TOOLS cleanSam tools (http://broadinstitute.github.io/picard.). Local realignment of insertions and deletions (indels) was then performed using the GATK suite [40] before final sorting and indexing. The RAMSES application (PMID: 24296945), written in-house, was used to detect nucleotide substitutions. Small indels were identified using Pindel [41] in paired tumor-normal mode. For greater specificity, only simple insertion and deletion events of fewer than 10 bp were selected. An in-house perl script filter was used to extract high-quality indels: considering the high sequence coverage obtained in these samples, only those indels with a minimum coverage of 20 reads in both tumor and normal samples, and with a minimum frequency of 10% of the reads and a minimum of five independent reads supporting the event in the tumor sample, and with no evidence in the normal sample, were considered. All potential somatic mutations were filtered using the dbSNP132 and 1000 Genomes Project mutation databases and the functional consequence at the protein level was annotated according to the Ensembl database using an in-house perl script based on the Ensembl database API.

### Validation analysis

Genomic DNA was amplified using the specific oligonucleotides described in Supplementary Table IV. All amplicons from the same patient were mixed in a tube and each sample was quantified by Qubit 2.0 (Life Technologies), using the Qubit^®^ dsDNA BR Assay Kit (Life Technologies). MELANOMA17 cells were monitored by Sanger sequencing for the presence of mutations in *BRAF* and *MAPK7* (see the supplementary methods for further details).

### Statistics

Unless otherwise specified, all experiments were done in independent triplicates and all numerical data were summarized as the average of the values ± the standard error of the mean (SEM) using GraphPad PRISM. Levels of statistical significance are indicated as follows: *p < 0.05; **p < 0.01; ***p < 0.001.

### Western blot

Cells growing exponentially at approximately 70% confluence were treated under the desired conditions. Cells were starved overnight (unless otherwise stated), treated with the appropriate inhibitor and lysed as described in [42]. Whole cell lysates were subjected to acrylamide SDS-PAGE, using standard procedures, then transferred onto a nitrocellulose support membrane (Immobilon, Millipore) and western blotted. The primary and secondary antibodies and the data collection method are described in the Supplementary Methods.

### Mice and reagents for *in vivo* studies

BALB/c Nude mice CAnN.Cg-Foxn1nu/Crl (Charles River) were injected with 6 × 10^6^ A375 melanoma cells in the subcutaneous dorsal area. Approximately one week later, the tumor reached a volume of about 100 mm^3^, at which point mice were assigned to four tumor size-comparable groups of 12 animals and treated as described in the Supplementary Methods.

Fresh tissue from patient 17, who had been diagnosed with metastatic melanoma (Table I and Figure 5), was minced and xeno-injected into NOD.Cg-Prkdcscid Il2rgtm1Wjl/SzJ mice (commonly known as NOD scid gamma (NSG) mice) (Charles River). Briefly, the animals were anesthetized using ketamine (75 mg/kg) and medetomidine (1.0 mg/kg) and a piece of tumor was inserted in the subcutaneous dorsal area through a small incision in the skin and allowed to grow. Next, mice were sacrificed (as described in supplementary methods) and tumors were collected and minced into pieces of about 2 mm^3^ and reimplanted into the experimental group of mice. When these mice had grown tumors of an approximate volume of 100 mm^3^, they were distributed among four groups of six mice, each with comparable tumor volumes and treatments were started, as described in Figure 5 and in the Supplementary Methods.

## SUPPLEMENTARY MATERIALS AND METHODS










