# Erratum: Individualized strategies to target specific mechanisms of disease in malignant melanoma patients displaying unique mutational signatures

**DOI:** 10.18632/oncotarget.7118

**Published:** 2016-02-01

**Authors:** Soraya Curiel-Olmo, Almudena García-Castaño, Rebeca Vidal, Helena Pisonero, Ignacio Varela, Alicia León-Castillo, Eugenio Trillo, Carmen González-Vela, Nuria García-Diaz, Carmen Almaraz, Thaidy Moreno, Laura Cereceda, Rebeca Madureira, Nerea Martinez, Pablo Ortiz-Romero, Elsa Valdizán, Miguel Angel Piris, José Pedro Vaqué

Present: Due to a technical error, the following author names were indexed incorrectly in PubMed: Piris MA and Vaqué JP

Correct: The abbreviation has been updated to reflect correct spelling of these authors' names. The publisher sincerely apologizes for this oversight

Original Article: Oncotarget. 2015; 6: 25452-25465. doi: 10.18632/oncotarget.4545.

