# Supplementary material for: Individualized strategies to target specific mechanisms of disease in malignant melanoma patients displaying unique mutational signatures
Source: Oncotarget. 2015 Jul 25;6(28):25452–65. doi: 10.18632/oncotarget.4545 (PMC4694844; doi:10.18632/oncotarget.4545)
Supplement: Supplementary file 1 [file oncotarget-06-25452-s001.pdf]

SUPPLEMENTARY MATERIALS AND METHODS

CELLS AND REAGENTS FOR TISSUE CULTURE

Commercial cell lines were cultured as recommended by ATCC or DSMZ. MELANOMA17 cells were cultured in DMEM medium (Lonza, Basel Switzerland) supplemented with 10% heat-inactivated fetal bovine serum (FBS) (Life Technologies), glucose (4.5 g/L), L-glutamine (292 mg/L), streptomycin sulfate (10 mg/L) and potassium penicillin (10000 U/L) (Lonza). All cell lines used are adherent cells, which were cultured at 37°C in a humidified sterile atmosphere of 95% air and 5% CO_2.

To perform functional analysis all inhibitors used in this study were obtained from Selleck Chemicals (Houston, TX): Vemurafenib (PLX4032), Vargatef (BIBF1120), Everolimus (RAD001), Ruxolitinib (INCB018424), Selumetinib (AZD6244), XMD8–92, Dacomitinib (PF299804, PF299), Fasudil (HA-1077) HCl, Crizotinib (PF-02341066), Tacrolimus (FK506), Imatinib (STI571) and Zibotentan (ZD4054). These drugs were reconstituted in DMSO and kept at -20°C until use.

MELANOCYTE ISOLATION AND CHARACTERIZATION

A patient-derived cell line (MELANOMA17) was established from a freshly resected tumor biopsy from patient17 (diagnosed with advanced melanoma, see Table 1), as described previously [1]. Briefly, tumor tissue was minced using sterile crossed blades under sterile conditions. The pieces were plated sparsely in 6-well tissue culture plates covered in culture medium. Plates were incubated in a humidified sterile atmosphere at 37°C and 5% CO_2. When cells emerging from tissue pieces became confluent, they were passaged at 1:2 dilution. After isolation, cells were labeled with anti-melanoma (MCSP) antibody conjugated with PE (Miltenyi Biotec, 130-099-413), which recognizes the melanoma-associated chondroitin sulfate proteoglycan (MCSP), and analyzed by FACS, using the appropriate controls (supplementary figure 5B). MCSP is expressed in the majority of human melanoma tissues and melanoma cell lines, but not in carcinoma, fibroblastoid or other cells of hematopoietic origin. In order to determine the melanocytic nature of MELANOMA17 cells, 10 µl of anti-MCSP-PE conjugated antibody were added to 8×10^6 cells in 100 µl of phosphate buffered saline (PBS). Following the manufacturer's instructions, after 10 min incubation in the dark at 4°C, cells were washed and centrifuged at 300 *g* for 10 min. The supernatant was completely aspired and the cell pellet resuspended in 500 µl. Finally, cells were analyzed by FACS, collecting data from 10,000 cells on a BD FACSCanto™ II flow cytometer (BD Biosciences). Data were analyzed using BD FACSDiva™ Software (BD Biosciences).

CELL PROLIFERATION AND DNA SYNTHESIS ASSAYS

Cells were seeded in a 96-well plate at a density of 3,000 cells per well overnight at 37°C (with 10% CO_2 and at 96% RH) unless otherwise stated. After that time, cells were attached and exponentially grown to approximately 50% confluence, and the appropriate concentrations of inhibitors were added in each case to the medium, using eight concentrations and keeping the total amount of DMSO (0.5%) constant under all conditions. Cellular proliferation was evaluated using alamarBlue reagent (Life Technologies), following the manufacturer instructions, 0, 24 and 48 h after addition of the drugs. Colorimetric changes were quantified by using the Synergy™ HTX Multi-Mode Microplate Reader (Biotek). IC_{50} values were obtained using GraphPad Prism 5 software (GraphPad Software Inc., La Jolla, CA, USA). To assess the effects on DNA synthesis, 10,000 cells per well were seeded in a Millicell EZ SLIDE 8-well glass (Merck Millipore, PEZGS0816) and incubated until cells were attached and grown to approximately 50% confluence. Inhibitors were then added using specific IC_{50} concentrations in each case. At these settings, cells were treated for 48 h and incubated for a further 2 h with Click-iT® EdU (Alexa Fluor® 594 Imaging Kit; Life Technologies, C10339) following the manufacturer's specifications. Immediately afterwards, cells were fixed using 3.7% formaldehyde in PBS and permeabilized with 0.5% Triton X-100. Finally, DNA was stained using Hoechst 33342 1:2000 in PBS. Cell images were captured with a Nikon A1R confocal microscope with Plan Apo 10x/0.45NA and Plan ApoVC 60x/1.40NA objectives. A 405-nm laser diode was used to excite Hoechst 33342 and a 561-nm laser diode was used to excite Alexa Fluor 594. Images were processed and analyzed using the object count tool of Nis elements software. Briefly, red and blue nuclei were segmented, channels were separated, and all images thresholded with the same parameters. Finally, the fluorescence of the segmented nuclei was measured.

GENOMIC DNA SAMPLES QUALITY TEST

Genomic DNA was extracted from fresh and/or frozen tissue by the phenol-chloroform method. The quantity and integrity of purified DNA was assessed by

Qubit 2.0 fluorometric quantitation (Life Technologies) and by capillary electrophoresis in a 2100 Bioanalyzer (Agilent Technologies) following the manufacturer's standardized instructions.

VALIDATION ANALYSIS

Genomic DNA was amplified using the specific oligonucleotides described in supplementary Table IV. All amplicons from the same patient were mixed in a tube and each of these samples was quantified by Qubit 2.0 (Life Technologies), using the Qubit® dsDNA BR Assay Kit (Life Technologies). 500 ng of each DNA sample was repaired using NEBNext: Ultra End Repair/dA Tailing Module kit (Biolabs) and linked to a pair of adapters; 3′-end and 5′-end, respectively. Then, a pair of indexing primers was bound to the adapters to allow subsequent identification of each sample. DNA was purified with Agencourt AMPure XP beads (Beckman Coulter) and 4 ng of each DNA was sequenced by Next Generation Sequencing, using a MiSeq Personal platform (Illumina). MELANOMA17 cells were monitored by Sanger sequencing for the presence of the following mutations by amplification using the following oligonucleotides: BRAF-V600E: Fw 5-AGCATCTCAGGGCCAAAAAT and Rv: 5-AACACATTTCAAGCCCCAAA; and MAPK7 (P546S): Fw 5-ATCTGCGGGCTCTACCTCTG and Rv 5-GACATGGAAGACTGAGGGGC. Each DNA was then amplified by PCR at Tm = 60°C, for 18 cycles, using a Herculase II Fusion Enzyme (Agilent Technologies) and analyzed by Sanger sequencing. We could not validate mutations in four patients due to technical difficulties (i.e., lack of sample or melanin strongly darkening the DNA).

STATISTICS

Unless otherwise specified, all experiments were done in independent triplicates and all numerical data were summarized as the average of the values ± the standard error of the mean (SEM) using GraphPad PRISM. *$p < 0.05$; **$p < 0.01$; ***$p < 0.001$.

WESTERN BLOT

Exponentially growing cells at approximately 70% confluence were treated under the desired conditions. Cells were starved overnight (unless otherwise stated), treated with the appropriate inhibitor and lysed as described in [2]. Whole cell lysates were subjected to acrylamide SDS-PAGE, using standard procedures, then transferred onto a nitrocellulose support membrane (Immobilon, Millipore) and western blotted. The primary antibodies, at 1:1000 dilution unless otherwise stated, were: anti-B-RAF (Rabbit B-Raf 55C6 IgG, Cell Signaling, Ref: 9433S), anti-FGFR2 (Rabbit FGFR2 D4H9 IgG, Cell Signaling, Ref: 11835S), P-ERK1/2 (Rabbit Phospho-p44/42 MAPK (T202/Y204) IgG, Cell Signaling, Ref: 4370S), ERK1/2 (Rabbit p44/42 MAPK 137FS IgG, Cell Signaling, Ref: 4695S), P-S6 (Rabbit Phospho-S6 Ribosomal protein (S235/236) D57.2.2E IgG, Cell Signaling, Ref: 4858S), S6 (Rabbit S6 Ribosomal protein 5G10 IgG, Cell Signaling, Ref: 2217S), P-p38 (Rabbit Phospho-p38 MAPK (T180/Y182) D3F9 IgG, Cell Signaling, Ref: 4511S), p38 (Rabbit p38 MAPK IgG2B, Cell Signaling, 8690S 8690BC), P-AKT (Rabbit Phospho-AKT (S473) IgG, Cell Signaling, Ref: 9271L), AKT (Rabbit AKT IgG, Cell Signaling, Ref: 9272), ERK5 (Rabbit ERK5 IgG, Cell Signaling, Ref: 3372S), P-STAT1 (Rabbit Phospho-STAT1 IgG, Cell Signaling, Ref: 9167S), STAT1 (Rabbit Stat 1 IgG, Cell Signaling, Ref: 9172S 9172P), S100 (Mouse S100 IgG, Cell Signaling, Ref: 5529S), mTOR (Rabbit mTOR IgG, Sigma Aldrich, Ref: SAB4501038), and anti-tubulin (Mouse α Tubulin (B-5-1-2) IgG, Santa Cruz Biotechnology, Ref: sc-23948). The secondary antibodies, all at 1:20,000 dilution, were: Goat-anti-mouse IgG, DyLight TM800 (Thermo Scientific, Ref: 35521) and goat-anti-rabbit IgG, DylightTM800 (Thermo Scientific, Ref: SA5–10036). Finally, data were collected using an Odyssey Infrared imaging system (Li-Cor).

IMMUNOHISTOCHEMISTRY

Immunohistochemical (IHC) expression of P-ERK1/2 and Ki67 was assessed using routine IHC techniques for five tumoral samples from mice bearing tumors. IHC staining was performed on tissue microarray sections using the following antibodies: Ki67 (clone MIB-1, Dako, Ref M7420), NFATc1 (clone 7A6, BD Biosciences, Ref 556602) and P-ERK1/2 (Rabbit Phospho-p44/42 MAPK (T202/Y204) IgG, Cell Signaling, Ref: 43765). The percentage of positive cells was determined in five fields from each tumor.

MICE AND REAGENTS FOR *IN VIVO* STUDIES

BALB/c Nude mice CAnN.Cg-Foxn1nu/Crl (Charles River) were injected with 6×10^6 A375 melanoma cells each in the subcutaneous dorsal area. Approximately one week after injection, the tumor reached a volume of about 100 mm^3, at which point treatments were started. At this time, mice were distributed among four tumor size-comparable groups of 12 animals: A) Vehicle, B) Vemurafenib (BRAFi (V)): 50 mg/kg, once a day, by oral gavage, C) Dual treatment with Everolimus (mTORi(E)): 10 mg/kg, once a day by oral gavage together with Vargatef (FGFR2i(Va)): 10 mg/kg, once a day by ip, and D) a triple treatment with Vemurafenib (BRAFi(V)), Everolimus (mTORi(E)) and Vargatef (FGFR2i(Va))

under the same conditions as above. Periodically, mice were weighed to detect any changes in weight during the experiment and tumor size was measured with an electronic caliper (Mitutoyo), to obtain tumor growth data. After 13 days of treatment, mice were sacrificed by cardiac puncture and exsanguination, after administering 75 mg/kg ketamine, ip, and 1.0 mg/kg medetomidine, ip, as anesthesia. Tumors were collected and divided into two halves, one of which was fixed in 4% formaldehyde and the other was frozen and stored at −80°C. Blood plasma was obtained in a density gradient medium, Lymphoprep (Stemcell Technologies), and stored at −80°C.

Fresh tissue from patient17 diagnosed with metastatic melanoma (see Table 1 and figure 5) was minced under sterile conditions in pieces of about 2 mm^3 and xenoinjected into NOD.Cg-Prkdcscid Il2rgtm1Wjl/SzJ mice, commonly known as the NOD scid gamma (NSG) (Charles River). Briefly, mice were anesthetized using ketamine (75 mg/kg) and medetomidine (1.0 mg/kg) and a piece of tumor was placed in the subcutaneous dorsal area through a small incision in the skin until it had grown. Next, mice were sacrificed as described above and tumors were collected and minced into pieces of about 2 mm^3 each and replanted in the experimental group of mice. When these mice had grown tumors with an approximate volume of 100 mm^3, they were distributed among four groups of 6 mice each with comparable tumor volumes and treatment was started. The first group was treated with vehicle twice a day; the second group was treated with Vemurafenib (50 mg/kg) once a day (by oral gavage); the third group received XMD8–92 (50 mg/kg) twice a day (ip); and the fourth group was given a double treatment: Vemurafenib and XMD8–92, under the same conditions as before. In all cases, manipulation and vehicle were kept constant. Periodically, mice were weighed to detect possible changes and the tumor size was measured using an electronic caliber (Mitutoyo) to collect tumor growth data. After 13 days of treatment mice were sacrificed (see above) and tumors were collected and divided into two halves, one of which was fixed in 4% formaldehyde and the other was frozen and stored at −80°C. Blood plasma was obtained in a density gradient medium, Lymphoprep (Stemcell Technologies) and stored at −80°C. The statistical significance of differences between groups was established by Student's independent samples *t*-test (SPSS v17.0).

REFERENCES

1. Turajlic S., et al. Whole-genome sequencing reveals complex mechanisms of intrinsic resistance to BRAF inhibition. Ann Oncol. 2014; 25:959–67.
2. Chiariello M., et al. Activation of Ras and Rho GTPases and MAP Kinases by G-protein-coupled receptors. Methods Mol Biol. 2010; 661:137–50.

SUPPLEMENTARY FIGURES AND TABLES

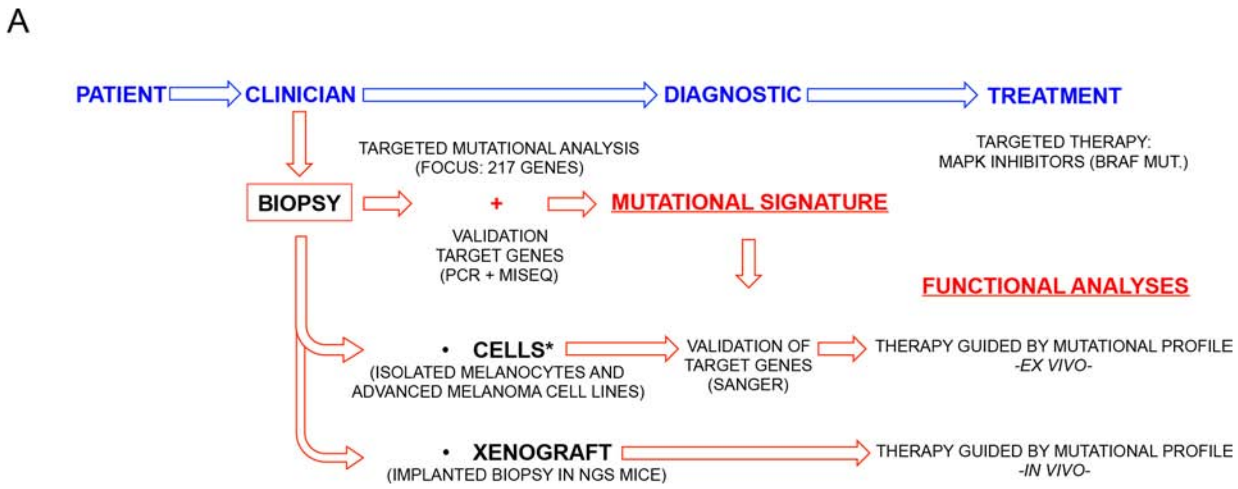

Supplementary Figure S1: Working pipeline scheme for this study. Blue line shows a normal clinical process towards the diagnosis of advanced melanoma. In red, we show our approach consisting of the generation of a validated mutational profile for each lesion that we can associate with inhibitors that can be tested in functional analyses: *ex vivo*, in well-established or patient-derived cells, or *in vivo*, using implanted biopsies growing in NSG mice.

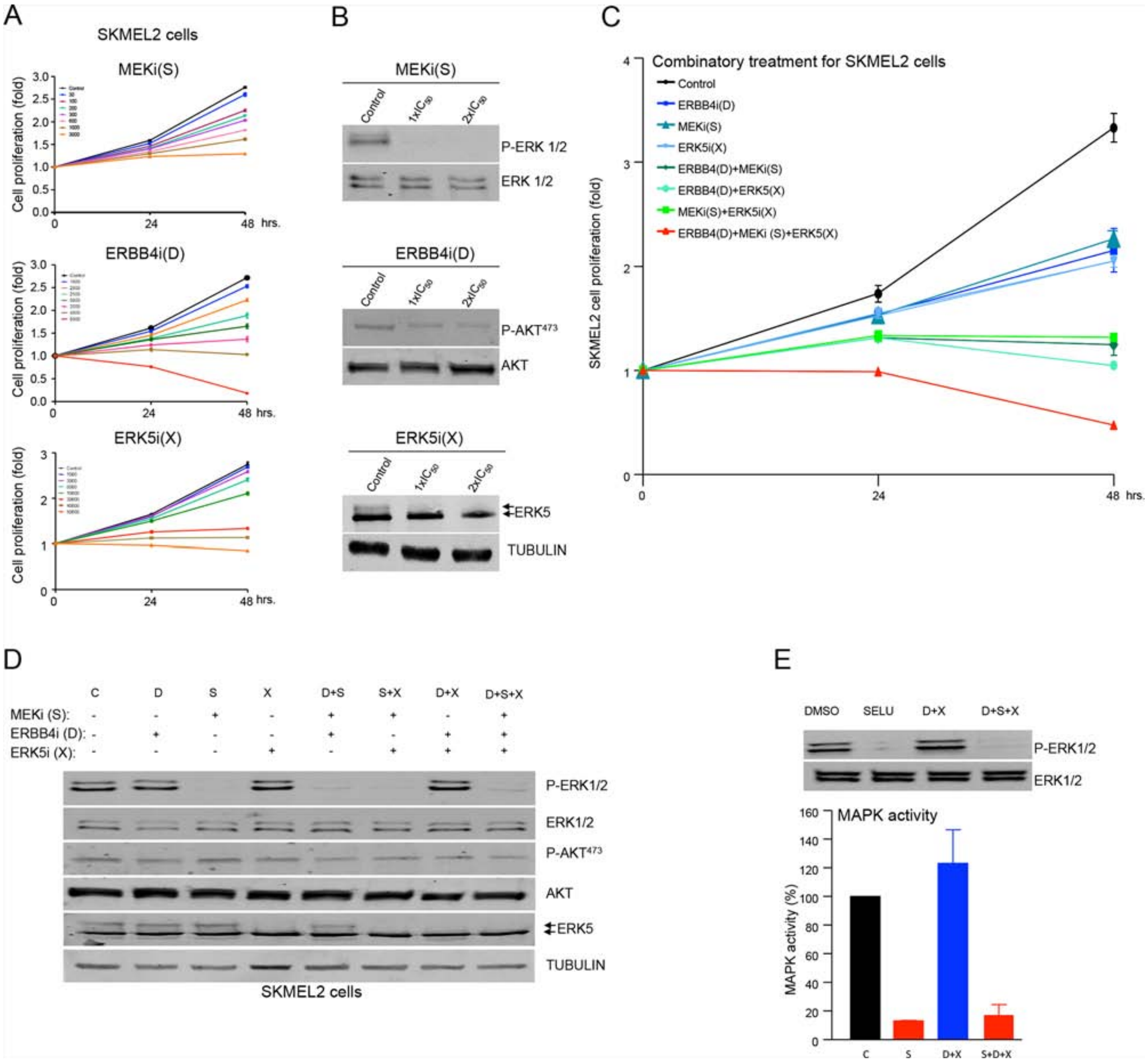

Supplementary Figure S2: Effects of a specific targeted therapy guided by mutational signature in SKMEL2 cells. A. Proliferation analysis of SKMEL2 cells at 0, 24 and 48 h. 3×10^3 cells/well were seeded in 96-well plates and treated with the indicated concentrations of each inhibitor: MEKi (S: Selumetinib), ERBB4 (D: Dacomitinib) or ERK5i (X: XMD-8–92). **B.** Western blots using whole cell lysates from starved SKMEL2 cells incubated for 1 h with control vehicle (DMSO) or the indicated concentration of each inhibitor. The figure shows a representative experiment using P-ERK1/2, ERK1/2, P-AKT^{473}, AKT, ERK5 and tubulin antibodies as indicated. **C.** Proliferation analysis of SMEL2 cells under the same conditions as in A) but incubated with control vehicle (DMSO) or the IC_{50} concentration of the indicated inhibitor alone (blue lines), in a double (green lines) or a triple combination (red line). $N = 6$. Error bars show SEM. **D** and **E.** Western blots using whole cell lysates of the indicated cells. Cells were starved overnight and incubated for 1 h with control vehicle (DMSO), or indicated inhibitor, or a combination of inhibitors under the same conditions as in C). Figures show representative experiments using the antibodies as indicated. Graph bars show values from three independent experiments in E). Error bars indicate SEM.

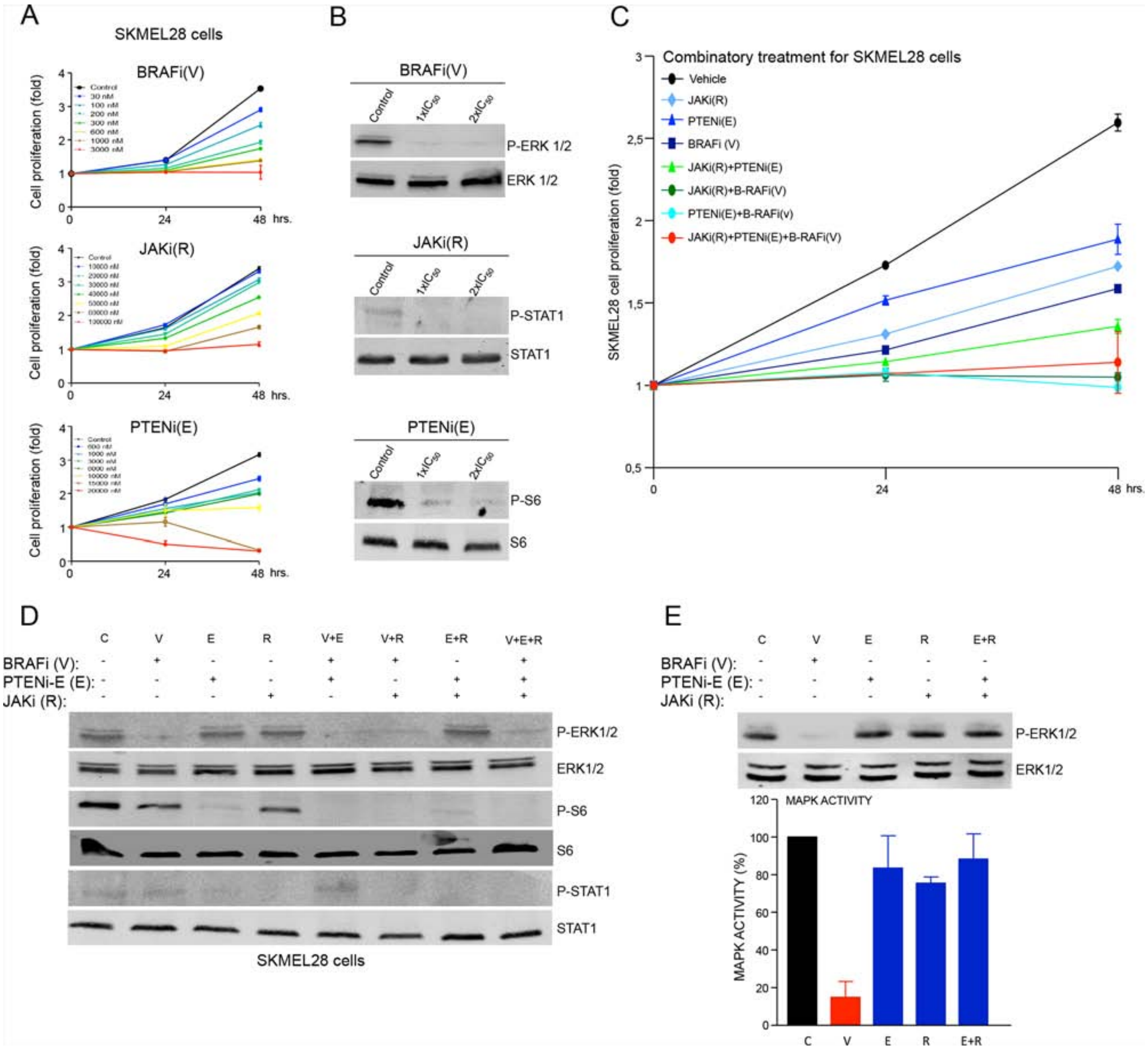

Supplementary Figure S3: Effects of a specific targeted therapy guided by mutational signature in SKMEL28 cells. A. Proliferation analysis of SKMEL28 cells at 0, 24 and 48 h. 3×10^3 cells/well were seeded in 96-well plates and treated with the indicated concentrations of each inhibitor: BRAFi (V: Vemurafenib), JAKi (R: Ruxolitinib) or PTENi (E: Everolimus). **B.** Western blots using whole cell lysates from starved SKMEL28 cells incubated for 1 h with control vehicle (DMSO) or the indicated concentration of each inhibitor. The figure shows a representative experiment using P-ERK1/2, ERK1/2, P-S6, S6, P-STAT1, and STAT1 antibodies, as indicated. **C.** Proliferation analysis of SMEL28 cells under the same conditions as in A) but incubated with control vehicle (DMSO) or the IC_{50} concentration of the indicated inhibitor alone (blue lines), in a double (green lines) or a triple combination (red line). $N = 6$. Error bars show SEM. **D** and **E.** Western blots using whole cell lysates of the indicated cells. Cells were starved overnight and incubated for 1 h with control vehicle (DMSO), or indicated inhibitor, or a combination of inhibitors under the same conditions as in C). Figures show representative experiments using P-ERK1/2, ERK1/2, P-S6, S6, P-STAT1 and STAT1 antibodies as indicated. Graph bars show values from three independent experiments in E). Error bars indicate SEM.

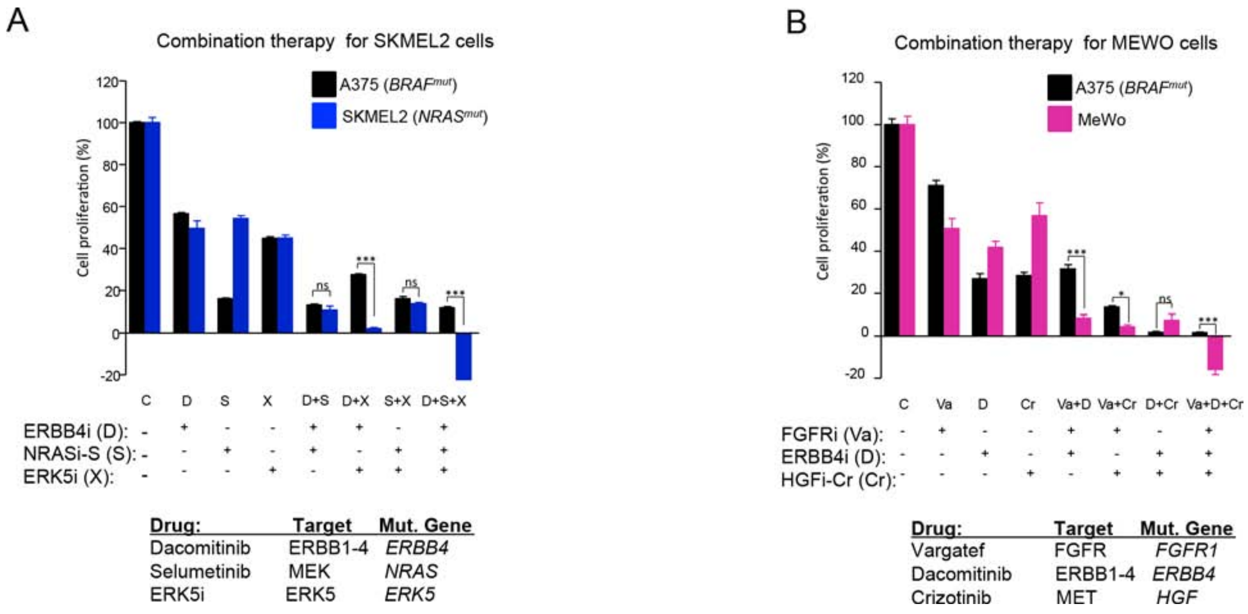

Supplementary Figure S4: Increased effect of a targeted therapy within an appropriate mutational background (2). A. Proliferation analysis of exponentially growing A375 (BRAF+) and SKMEL2 (NRAS+) cells. 3×10^3 cells/well were seeded in 96-well plates and treated with control vehicle (DMSO) or the IC_{50} concentration (calculated for SKMEL2 cells, see supplementary Table 1) of the indicated inhibitor alone, in a double or a triple combination for 48 h. $N = 6$. Error bars show SEM. **B.** Proliferation analysis of exponentially growing A375 (BRAF+) and MEWO cells. 3×10^3 cells/well were seeded in 96-well plates and treated with control vehicle (DMSO) or the IC_{50} concentration (calculated for MEWO cells; see supplementary Table 1) of the indicated inhibitor alone, in a twosome or a threesome combination for 48 hours. $N = 6$, error bars show SEM.

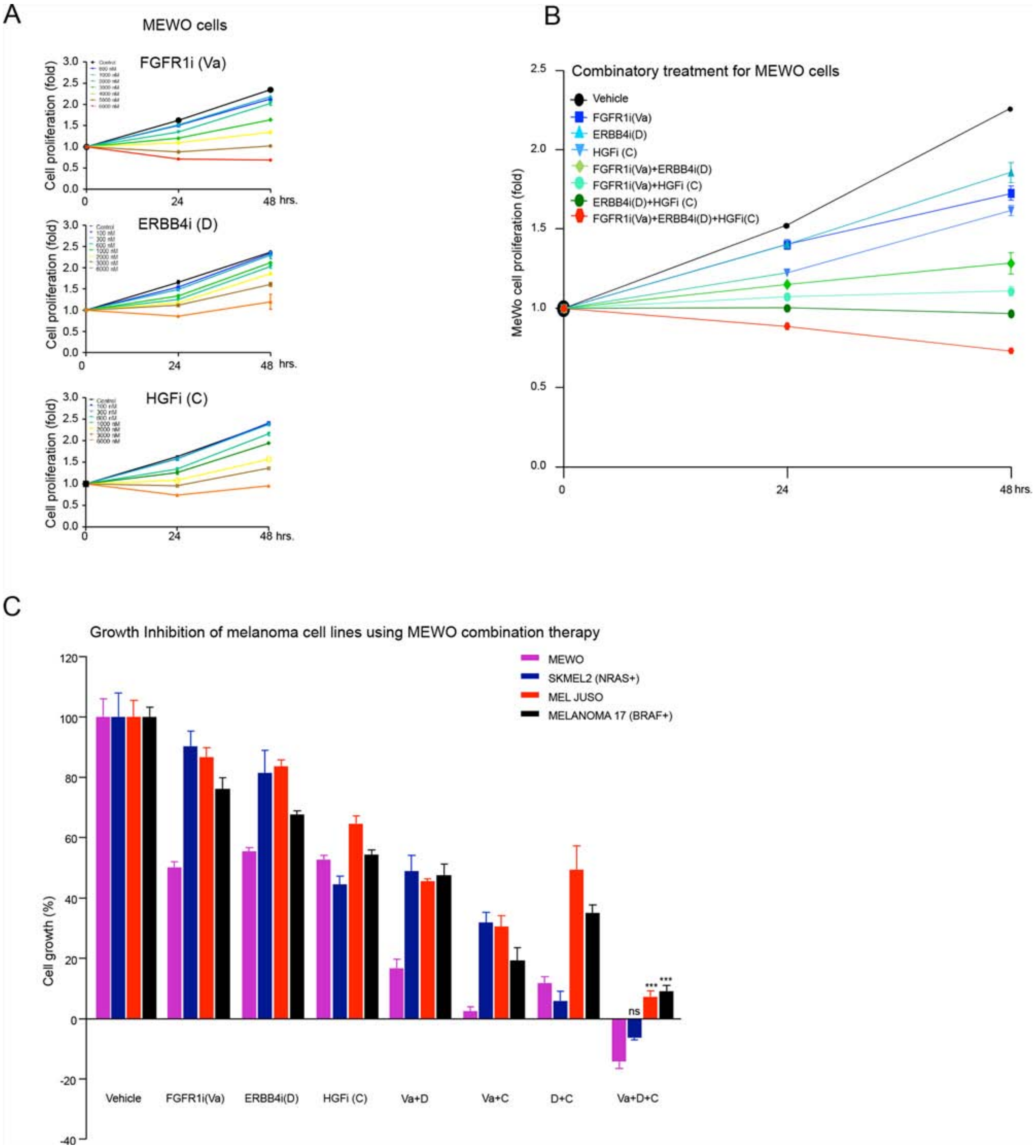

Supplementary Figure S5: Effects of specific targeted therapy guided by mutational signature in MEWO cells. Increased effect of a targeted therapy against an appropriate mutational background (3). A. Proliferation analysis of MEWO cells at 0, 24 and 48 h. 3×10^3 cells/well were seeded in 96-well plates and treated with the indicated concentrations of each inhibitor: FGFR1i (Va: Vargatef), ERBB4i (D: Dacomitinib) or HGFi (C: Crizotinib). **B.** Proliferation analysis of MEWO cells under the same conditions as in A) but incubated with control vehicle (DMSO) or the IC_{50} concentration (see supplementary Table 1) of the indicated inhibitor alone (blue lines), in a double (green lines) or a triple combination (red line). $N = 6$. Error bars show SEM. **C.** Proliferation analysis of exponentially growing MEWO, SKMEL2 (NRAS+), MEL JUSO (HRAS+) and MELANOMA17 (BRAF+) cells. 3×10^3 cells/well were seeded in 96-well plates and treated with control vehicle (DMSO) or the IC_{50} concentration (calculated for MEWO cells) of the indicated inhibitor alone, in a double or a triple combination for 48 hours. $N = 6$, error bars show SEM.

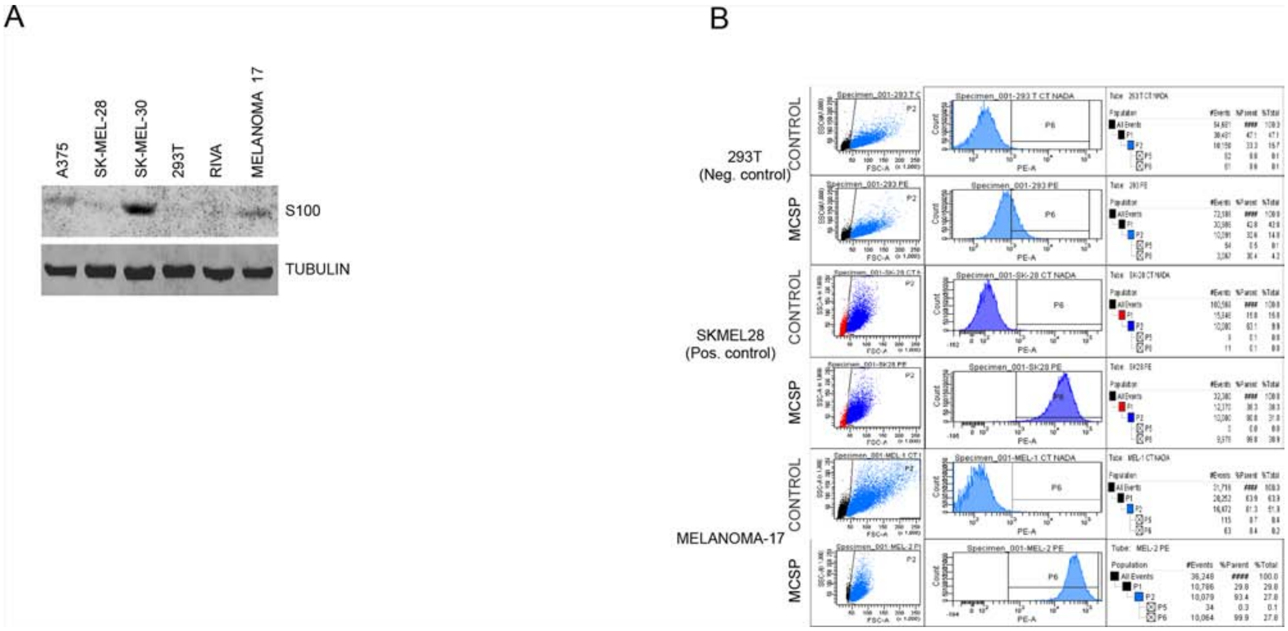

Supplementary Figure S6: Melanocytic characterization of cells isolated from melanoma patient-17. A. Western blot using whole cell lysates of the indicated cells. Figure show representative experiments using S100 and tubulin antibodies as indicated. **B.** FACS expression of MCSP in 293T (negative control), SKMEL28 (positive control) and MELANOMA17 cells incubated with anti-MCSP antibody. The figure shows a representative experiment.

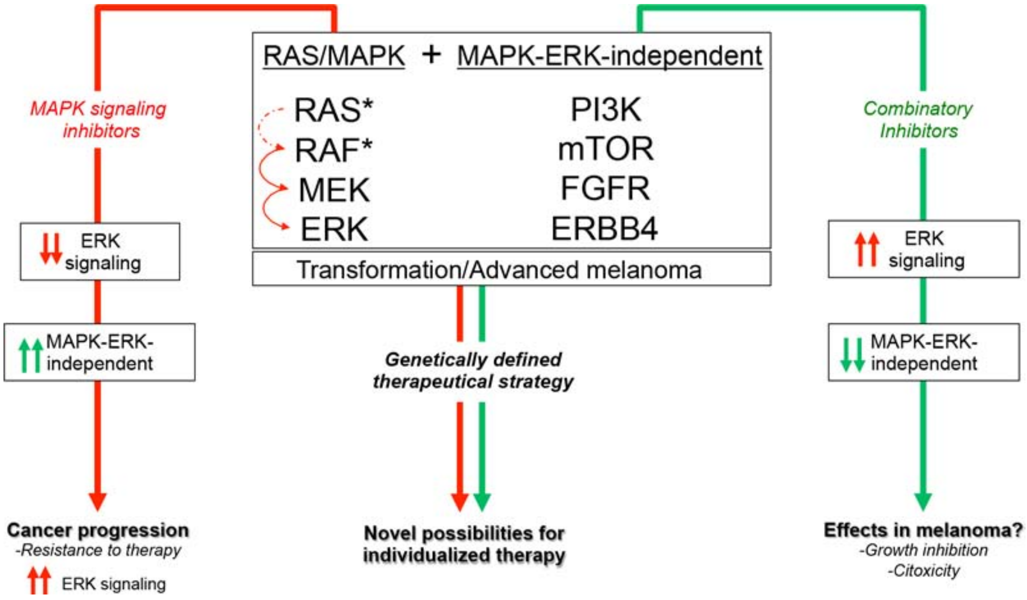

Supplementary Figure S7: Novel possibilities for targeting melanoma using combinatory therapies guided by mutational data. Current targeted therapy of advanced melanoma involves targeting aberrant MAPK-ERK signaling, guided by the detection of activating mutations in BRAF (red line). This produces a rapid but transient effect that ends in the development of multiple mechanisms of resistance that have been shown to reactivate MAPK signaling. Using the targeted approach proposed in this study we can detect mutations in genes that may participate in other potentially oncogenic signaling pathways that promote melanocytic transformation and disease progression (MAPK-independent). Targeting these pathways without affecting MAPK may also inhibit melanoma growth directly (*per se*) or indirectly (cytotoxic effects of aberrant MAPK signaling) (green line). Thus, we can design novel therapeutic strategies (genetically defined) that offer novel possibilities for targeted therapy in individually characterized advanced melanoma cases.

Supplementary Table SI: Potentially actionable mutations found *in silico* (refers to figure 1). GENE: mutated gene; PATHWAY: Signaling pathway and MT. NUMBER: Number of mutations detected in each gene.

Supplementary Table SII: Clinical data from patients analyzed with HaloPlex. The table shows the clinical characterization of the lesions from 18 advanced melanoma patients included prospectively in our mutational study: Age at diagnosis (Dx), sex, Breslow index (B.I.), location of sample, Clark level and diagnosis. N/D: Not determined.

Supplementary Table SIII: Potentially actionable mutations and related inhibitors in the melanoma cell lines. Table showing the mutational characteristics and the ex vivo treatment effects of 11 commercial cell lines (in silico comparison with CCLE data) and one melanoma cell line isolated from a biopsy from patient17 (ex vivo data obtained prospectively). The table includes the name of the mutated gene, mutation (showing aminoacid change), inhibitor name (used throughout this report), and inhibitor (general name). IC_{50} (μM): micromolar IC_{50} concentration. N/A: Not applicable. N/D: Not determined.

Supplementary Table SIV: Primers used for mutational validation. The table shows genes and genomic locations studied to validate mutations found in our primary analysis
